# Immune-Desert Tumor Microenvironment in Thoracic SMARCA4-Deficient Undifferentiated Tumors with Limited Efficacy of Immune Checkpoint Inhibitors

**DOI:** 10.1093/oncolo/oyac040

**Published:** 2022-03-12

**Authors:** Justine Gantzer, Guillaume Davidson, Bujamin Vokshi, Noëlle Weingertner, Antoine Bougoüin, Marco Moreira, Véronique Lindner, Guillaume Lacroix, Céline Mascaux, Marie-Pierre Chenard, François Bertucci, Irwin Davidson, Jean-Emmanuel Kurtz, Catherine Sautès-Fridman, Wolf H Fridman, Gabriel G Malouf

**Affiliations:** Department of Medical Oncology, Strasbourg-Europe Cancer Institute (ICANS), Strasbourg, France; Fédération de Médecine Translationnelle (FMTS), Strasbourg, France; Department of Cancer and Functional Genomics, INSERM UMR_S1258, Institute of Genetics and of Molecular and Cellular Biology, Illkirch, France; Department of Cancer and Functional Genomics, INSERM UMR_S1258, Institute of Genetics and of Molecular and Cellular Biology, Illkirch, France; Department of Cancer and Functional Genomics, INSERM UMR_S1258, Institute of Genetics and of Molecular and Cellular Biology, Illkirch, France; Fédération de Médecine Translationnelle (FMTS), Strasbourg, France; Department of Pathology, University Hospital, Strasbourg, France; Centre de recherche des Cordeliers, INSERM, Université de Paris, Sorbonne Université, Team 13- Complement, Inflammation and Cancer, Équipe labellisée Ligue contre le cancer, Paris, France; Centre de recherche des Cordeliers, INSERM, Université de Paris, Sorbonne Université, Team 13- Complement, Inflammation and Cancer, Équipe labellisée Ligue contre le cancer, Paris, France; Fédération de Médecine Translationnelle (FMTS), Strasbourg, France; Department of Pathology, University Hospital, Strasbourg, France; Centre de recherche des Cordeliers, INSERM, Université de Paris, Sorbonne Université, Team 13- Complement, Inflammation and Cancer, Équipe labellisée Ligue contre le cancer, Paris, France; Department of Pneumology, University Hospital, Strasbourg, France; University of Strasbourg, Inserm UMR_S 1113, IRFAC, Laboratory Streinth (STress REsponse and INnovative THerapy against cancer), Strasbourg, France; Fédération de Médecine Translationnelle (FMTS), Strasbourg, France; Department of Pathology, University Hospital, Strasbourg, France; Department of Medical Oncology, Cancer Research Center of Marseille (CRCM), INSERM U1068, CNRS UMR7258, Institut Paoli Calmettes, Aix-Marseille University, Marseille, France; Department of Cancer and Functional Genomics, INSERM UMR_S1258, Institute of Genetics and of Molecular and Cellular Biology, Illkirch, France; Department of Medical Oncology, Strasbourg-Europe Cancer Institute (ICANS), Strasbourg, France; Fédération de Médecine Translationnelle (FMTS), Strasbourg, France; Centre de recherche des Cordeliers, INSERM, Université de Paris, Sorbonne Université, Team 13- Complement, Inflammation and Cancer, Équipe labellisée Ligue contre le cancer, Paris, France; Centre de recherche des Cordeliers, INSERM, Université de Paris, Sorbonne Université, Team 13- Complement, Inflammation and Cancer, Équipe labellisée Ligue contre le cancer, Paris, France; Department of Medical Oncology, Strasbourg-Europe Cancer Institute (ICANS), Strasbourg, France; Fédération de Médecine Translationnelle (FMTS), Strasbourg, France; Department of Cancer and Functional Genomics, INSERM UMR_S1258, Institute of Genetics and of Molecular and Cellular Biology, Illkirch, France

**Keywords:** SMARCA4-deficiency, thoracic tumors, sarcomas, immunotherapy, immune infiltrate, tumor microenvironment

## Abstract

**Background:**

Thoracic SMARCA4-deficient undifferentiated tumors (SMARCA4-UT) are aggressive neoplasms. Data linking BAF alterations with tumor microenvironment (TME) and efficacy of immune checkpoint inhibitors (ICI) are contradictory. The TME of SMARCA4-UT and their response to ICI are unknown.

**Materials and Methods:**

Patients diagnosed with SMARCA4-UT in our institution were included. Immunostainings for tertiary lymphoid structures (TLS), immune cell markers, and checkpoints were assessed. Validation was performed using an independent transcriptome dataset including SMARCA4-UT, non–small cell lung cancers (NSCLC) with/without *SMARCA4* mutations, and unclassified thoracic sarcomas (UTS). CXCL9 and PD-L1 expressions were assessed in NSCLC and thoracic fibroblast cell lines, with/without *SMARCA4* knockdown, treated with/without interferon gamma.

**Results:**

Nine patients were identified. All samples but one showed no TLS, consistent with an immune desert TME phenotype. Four patients received ICI as part of their treatment, but the only one who responded, had a tumor with a TLS and immune-rich TME. Unsupervised clustering of the validation cohort using immune cell scores identified 2 clusters associated with cell ontogeny and immunity (cluster 1 enriched for NSCLC independently of *SMARCA4* status (*n* = 9/10; *P* = .001); cluster 2 enriched for SMARCA4-UT (*n* = 11/12; *P* = .005) and UTS (*n* = 5/5; *P* = .0005). *SMARCA4* loss-of-function experiments revealed interferon-induced upregulation of CXCL9 and PD-L1 expression in the NSCLC cell line with no effect on the thoracic fibroblast cell line.

**Conclusion:**

SMARCA4-UT mainly have an immune desert TME with limited efficacy to ICI. TME of SMARCA4-driven tumors varies according to the cell of origin questioning the interplay between BAF alterations, cell ontogeny and immunity.

## Teaser

Thoracic SMARCA4-deficient undifferentiated tumors (SMARCA4-UT) are aggressive neoplasms. This article reports the first comprehensive analysis on SMARCA4-UT immune infiltrate, focusing on the immune landscape and patient responses to immune checkpoint inhibition therapy.

**Implications for Practice**

Thoracic SMARCA4-deficient undifferentiated tumors harbor mostly an immune desert TME but, as in soft-tissue sarcomas, immune-rich tumors characterized by TLS do exist and respond to ICI. The TME of SMARCA4-driven tumors varies according to the cell of origin highlighting the need to explore the interplay between alterations of BAF complexes, cell ontogeny and immunity.

## Background

Thoracic SMARCA4-deficient undifferentiated tumors (SMARCA4-UT) are a rare type of neoplasm, characterized by inactivating *SMARCA4* mutations leading to protein loss.^1^ SMARCA4-encoded protein, BRG1 is one of the ATPase subunits part of the BRG1/BRM-associated factors (BAF) chromatin remodeling complex, also known as the mammalian SWItch/sucrose Non-Fermenting (mSWI/SNF) ATP-dependent chromatin remodeling complex.^2^ The BAF complexes, composed of multiple subunits, regulate transcription and recent studies revealed their critical roles as tumor suppressors.^3-5^ Gene profiling analyses revealed that SMARCA4-UT are related to other BAF complex-deficient tumors (ie, *SMARCB1*-inactivated malignant rhabdoid tumors (MRT) and *SMARCA4*-mutated-small cell carcinomas of the ovary, hypercalcemic type (SCCOHT)), but differ from SMARCA4-deficient non–small cell lung cancers (NSCLC) and not otherwise specified (NOS) NSCLC.^1^ Histologically SMARCA4-UT are poorly differentiated tumors with rhabdoid or epithelioid features and harbor a specific “immunohistochemical signature”. Indeed, in the fifth edition of World Health Organization (WHO) classification of Thoracic Tumors, the community of pathologists recognized this newly described entity but changed its name from SMARCA4-deficient thoracic sarcomas to SMARCA4-UT.^6^ Clinically, the majority of SMARCA4-UT shared an aggressive clinical course with a median overall survival (OS) between 4 and 7 months.^1,7-11^ Mostly male adults around 45 years old with a heavy smoking history are affected. Primary tumor location is thoracic with a median tumor size of 10 cm.^7^

Currently, there is no approved treatment for SMARCA4-UT. The first-line setting is mainly anthracycline-based chemotherapy identical to what is done for soft-tissue sarcomas (STS), but with very limited efficacy. Based on encouraging preclinical data, immune checkpoint inhibitors (ICI), especially anti-programmed cell death protein 1 (PD1), have been tested in STS patients, but showed limited efficacy with observed objective response rates always below 20%, although exceptional responders were identified.^12-15^ This limited efficacy has been explained by the analysis of tumor microenvironment (TME) composition, based on the study of the immune populations from the transcriptome of 213 STS.^16^ Notably, only 2 out of the 5 immune subtypes described harbored an immune-high phenotype despite a low tumor mutational burden (TMB). Within these subtypes, the one with a high response rate to ICI and an improved survival was characterized by the presence of tertiary lymphoid structures (TLS) in tumors. TLS are ectopic lymphoid formations structured with a T-cell zone with mature dendritic cells, a germinal center with proliferating B cells and are the lymphoid organs closest to the tumor generating an adaptative immune response.^17-19^ Other biomarkers of response to ICI, also described in STS, include higher densities of cytotoxic tumor-infiltrating T cells, activated T cells, and an increased percentage of tumor-associated macrophages expressing PD1 ligand 1 (PD-L1).^20^

Previous data from the literature regarding the link between alterations of BAF complexes and their correlation with immune infiltrate are contradictory.^21^ Indeed, while some studies have reported that BAF deficiency itself may enhance tumor cell susceptibility to immune control, others suggested that it may lead to impaired interferon (IFN) signaling leading to a non-immunogenic phenotype.^22-25^ Focusing on SMARCA4-deficient tumors, the first report of SMARCA4-deficiency TME was described in SCCOHT and unraveled an immune-active TME despite low TMB, with significant levels of T-cell infiltration and PD-L1 expression.^26^ Interestingly, among 4 patients treated with ICI, 1 patient had a sustained partial response for 6 months, and the 3 remaining patients remained disease-free for 1.5 years or more.^27^ Other promising results on the efficacy of ICI were published on different SMARCA4-deficient tumors,^28,29^ while others suggested the opposite.^30^ Recently, results of a phase II study assessing the efficacy of pembrolizumab in patients with rare sarcomas described 3 out of the 11 patients (27%) with SMARCA4-deficient MRT responding to ICI,^15^ which was consistent with several previously published case reports responding to ICI combined or not to chemotherapy.^31-34^ However, to our knowledge, the immune landscape of SMARCA4-UT remains unknown and there is no series describing patient’s response to ICI.

Herein, we report the first comprehensive analysis on SMARCA4-UT immune infiltrate, based on a retrospective cohort of 9 cases from a single institution and validate our findings in an independent cohort.^1^ We also describe the response of 4 patients to ICI and performed a temporal comprehensive genomic as well as immune tumor profiling of an exceptional responder with complete and lasting response to ICI. Finally, CXCL9 chemokine and PD-L1 expressions were assessed in NSCLC and thoracic fibroblast cell lines, treated with/without interferon gamma (IFNG).

## Materials and Methods

### Patients and Samples

Patients of Strasbourg University hospital were identified prospectively during the period of 2016 to 2019. The main clinicopathological data and outcomes were recorded. Sample collection for further research analysis was approved by an Ethical Committee (“Comité de Protection des Personnes Est IV”, Strasbourg, France) and the study was performed according to the Declaration of Helsinki.

### Immunohistochemistry and Immunofluorescence

Immunostainings carried out on diagnostic purposes were performed by the local expert sarcoma pathologist, according to routine practice (Supplementary Table S1). Immunostainings for immune cell markers (CD3, CD8, CD20, and CD68) and immune checkpoints (PD1, PD-L1, and TIM3) were performed and evaluated according to the methodology detailed in the Supplementary Appendix and in the list of antibodies (Supplementary Table S2).

Tumors were considered TLS positive when a CD3 aggregate was found juxtaposing a CD20 aggregate, as previously described.^19^ TLS with surface above 60 000 μm^2^, containing at least 700 cells were considered as mature when a network of follicular dendritic cells was detected in B cell follicle on haematoxylin and eosin slides.^35,36^ In lymph node metastases, TLS were only taken into account if the sample was invaded by more than 85% of tumor cells and without any residual lymph node tissue. PD-L1 was quantified using a score from 0 to 2 for both immune and tumor cells; 0 when no staining or less than 5% of positive cells, 1 between 5% and 50%, and 2 over 50% of positive cells.

For 7 patients, studied samples were either from the primary tumor (*n* = 2) or from distant metastasis (*n* = 5); for 2 patients, samples were available in both the primary tumor and matched distant metastasis.

### Comprehensive Genomic Profiling

A FoundationOne Heme test was performed for 2 distinct samples in patient no. 3. This available commercial genomic profiling test from Foundation Medicine sequences DNA and RNA to detect cancer-related genomic alterations in more than 400 genes and around 250 gene fusions. Microsatellite status and TMB were also assessed.

### Gene Expression Analysis

Publicly available fastq files of gene expression profiling from SMARCA4-UT (*n* = 12), SMARCA4-deficient NSCLC (*n* = 4), NOS NSCLC (*n* = 10), and unclassified thoracic sarcoma (UTS; *n* = 5) were downloaded from Sequence Read Archive accession SRP052896.^1^ Briefly, raw reads were aligned using STAR v2.5.3a with the “--quantMode TranscriptomeSAM” argument and by providing the GFF file from ENSEMBL v75, gene expression level was then calculated using RSEM v1.3.3.^37,38^ Gene expression data were processed using R v3.6.3. The TME composition of each sample was inferred using the microenvironment cell populations (MCP)-counter v1.2.0,^39^ providing abundance score for 8 immune populations and 2 stromal populations (fibroblast and endothelial cells) based on analysis of specific transcriptomic markers expressed by each cell population.^16^ We removed the fibroblast signature since sarcomas highly expressed this signature because of their mesenchymal origin, in order to better see differences in the other signatures. Samples were clustered based on their MCP-counter analysis using an unsupervised hierarchical clustering on the metagene *Z*-score for the 8 immune populations, with complete linkage using the hclust function and visualized as a heatmap with the pheatmap package v1.0.12.

Gene signatures for the functional orientation were computed as the geometric mean expression of the following genes: immunosuppression (*CXCL12*, *TGFB1*, *TGFB3*, and *LGALS1*), T-cell activation (*CXCL9*, *CXCL10*, *CXCL16*, *IFNG*, and *IL15*), T-cell survival (*CD70* and *CD27*), regulatory T cells (*FOXP3* and *TNFRSF18*), major histocompatibility complex class I (*HLA-A*, *HLA-B*, *HLA-C*, *HLA-E*, *HLA-F*, *HLA-G*, and *B2M*), myeloid cell chemotaxis (*CCL2*), tertiary lymphoid structures (*CXCL13*). Expression of genes related to immune checkpoint were also computed (*PD1*, *PD-L1*, *PD-L2*, *CTLA4*, *TIM3*, and *LAG3*).

### In vitro Analyses

Two commercially available cell lines were purchased from ATCC: the CCD_19Lu derived from human thoracic fibroblasts and the Calu-1 derived from NSCLC pleural metastasis. CCD_19Lu cells were grown in EAGLE medium supplemented with 10% FCS and gentamicine, and Calu-1 in MEM alpha medium with ribonucleosides and deoxyribonucleosides supplemented with 10% FCS and gentamicine. In the setting of this study, each cell line was transfected transiently with siRNAs either targeting SMARCA4 or non-targeting control for 6 hours, followed by either addition of DMSO as a control or human recombinant IFNG (20 ng/mL, Peprotech 500-P32). Cells were collected after 24 hours for RNA and protein extractions.

RNA isolation was performed according to standard procedure (NucleoSpin RNA Plus Kit). RT-qPCR was carried out with SYBR Green I (Roche) and SuperScript IV Reverse Transcriptase (Invitrogen) and monitored using a LightCycler 480 (Roche). The mean of *ACTB* gene expression levels was used to normalize the results. Primer sequences for each cDNA were designed using Primer3 Software and are available upon request.

Whole cell extracts were prepared by the standard freeze-thaw technique using LSDB 500 buffer (500 mM KCl, 25 mM Tris at pH 7.9, 10% glycerol (v/v), 0.05% NP-40 (v/v), 16 mM DTT, and protease inhibitor cocktail). Cell lysates were subjected to SDS–polyacrylamide gel electrophoresis (SDS-PAGE) and proteins were transferred onto a nitrocellulose membrane. Membranes were incubated with primary antibodies in 5% dry fat milk and 0.01% Tween-20 overnight at 4 °C. The membrane was then incubated with HRP-conjugated secondary antibody (Jackson ImmunoResearch) for 1 hour at room temperature, and visualized using the ECL detection system (GE Healthcare). The antibodies used are as follows: SMARCA4 (ab110641, Abcam), VCL (V4505, Sigma-Aldrich), and PD-L1 (13684, Cell Signaling).

### Statistical Analysis

Analysis of expression profiles was performed using Fischer’s exact test for categorical variable using Prism v7.0. Overall survival was analyzed with Kaplan-Meier estimates. All tests were 2-sided with a significative *P*-value defined <.05.

## Results

### Population Characteristics

**Figure 1.**
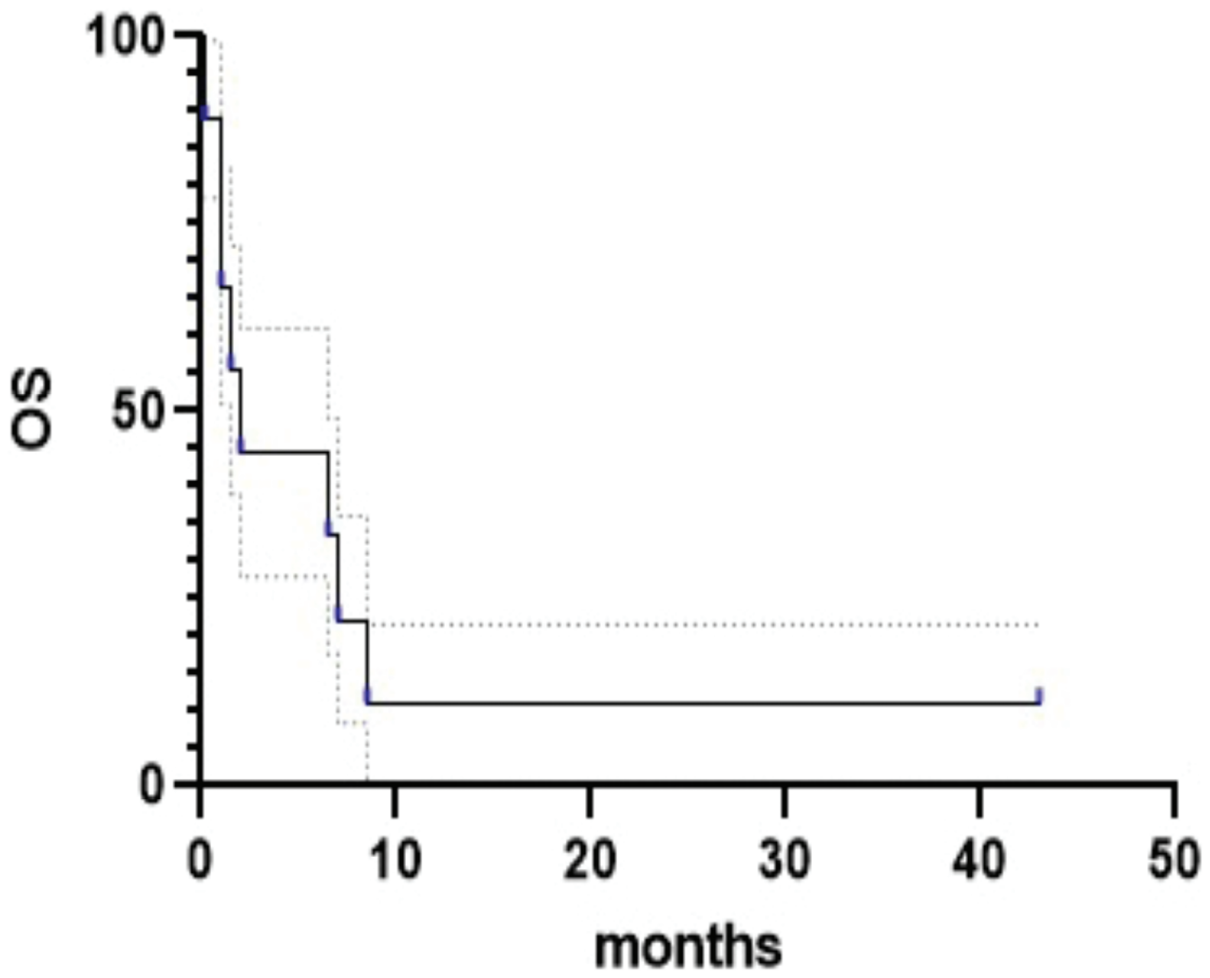
Kaplan-Meier overall survival (OS) curves for all 9 patients.

We studied the immune landscape of 11 SMARCA4-UT samples (total of 9 patients), including 4 primary thoracic tumors (2 biopsies and 2 surgical specimens) and 7 distant metastases: bone (*n* = 1), lymph node (*n* = 4), brain (*n* = 1), and jaw (*n* = 1). For 2 patients (no. 3 and no. 5), tumor specimens were available from paired primary and distant metastasis, and at different timepoints for patient no. 3. Patient and sample characteristics are described in Table 1. The median age was 60 years (range, 39-73 years) and most of them were male (67%) with a poor performance status. Primary tumor locations were well balanced with 5 and 4 tumors located in the mediastinum and lungs, respectively. Median tumor size was 8.5 cm (range, 4.2-16.9). All patients displayed metastatic disease at diagnosis with 78% and 45% of them with lymph node and bone metastasis, respectively. As first-line treatment, 5 patients (55%) received chemotherapy and 2 patients received ICI (one received nivolumab alone and the other one the association nivolumab-ipilimumab), whereas 2 received only best supportive care. There was no statistical difference between median progression-free survival of patients treated by chemotherapy (2 months) versus ICI (0.5 months) (*P* = .12). The median OS was 1.8 months (range, 0.2-not reached) (Fig. 1). Eight patients died of the evolution of their disease and only one is still alive without evidence of disease.

**Table 1.** Clinical characteristics of a cohort of patients with a thoracic SMARCA4-deficient undifferentiated tumor.

Pt	Age (years)	Sex	Primary tumor site (size)	Comorbidity	Presenting symptoms (OMS)	Specimen type (specimen site)	Sync. mets	Treatment	Outcome	Time to death (months)
1	63	M	Lung (7.5 cm)	Smoking	DGC, pain from bone mets (2)	Core biopsy (bone mets)	Liver, AG, bone	CT no response on 2 regimens, TT no response	DOD	7
2	73	F	Mediastinum (10 cm)	Diabetes AVC	DGC, chest pain, dysphagia (3)	Core biopsy (primary mediastinal tumor)	Lung, bone, LN	BSC	DOD	1
3	39	M	Lung (NA)	Smoking	DGC, jaw mass (2)	Surgical specimen (jaw mets) + surgical specimen (primary lung tumor)	Jaw	Surgery of jaw mets, CT no response on 2 regimens, ICI response, surgical resection of residual lung mass, adjuvant ICI	Alive	NA
4	54	M	Lung (NA)	Smoking	Neurologic disorders (2)	Surgical specimen (brain mets)	Brain	CT no response	DOD	1
5	40	M	Mediastinum (4.8 cm)	Smoking	Dyspnea, chest pain (2)	Core biopsy (LN mets)+ surgical specimen (primary mediastinal tumor)	LN	ICI no response	DOD	1.2
6	56	M	Mediastinum (9.5 cm)	Smoking, CLL	DGC, dyspnea (2)	Core biopsy (primary mediastinal tumor)	Lung, LN	CT no response	DOD	1.5
7	60	F	Mediastinum (16.9 cm)	Smoking	DGC, dyspnea (3)	Core biopsy (LN mets)	Bone, LN, AG	BSC	DOD	0.2
8	66	F	Mediastinum (NA)	Smoking	DGC, cervical mass (3)	Surgical specimen (LN mets)	Bone, LN, AG	ICI no response	DOD	2.2
9	69	M	Lung (4.2 cm)	Smoking, Lung cancer	Hemoptysis (2)	Fine needle aspiration (LN mets)	LN	CT no response, ICI no response	DOD	6.5

Abbreviations: AG, adrenal glands; BSC, best supportive care; CLL, chronic lymphocytic leukemia; CT, chemotherapy; DGC, deterioration of general condition; DOD: dead of disease; F, female; ICI, immune checkpoint inhibitor; L, line; LN: lymph nodes; M, male; mets: metastasis; NA, not available; Sync., synchronous; TT: targeted therapy; yrs, years.

### Characterization of SMARCA4-UT Immune Infiltrate

**Figure 2.**
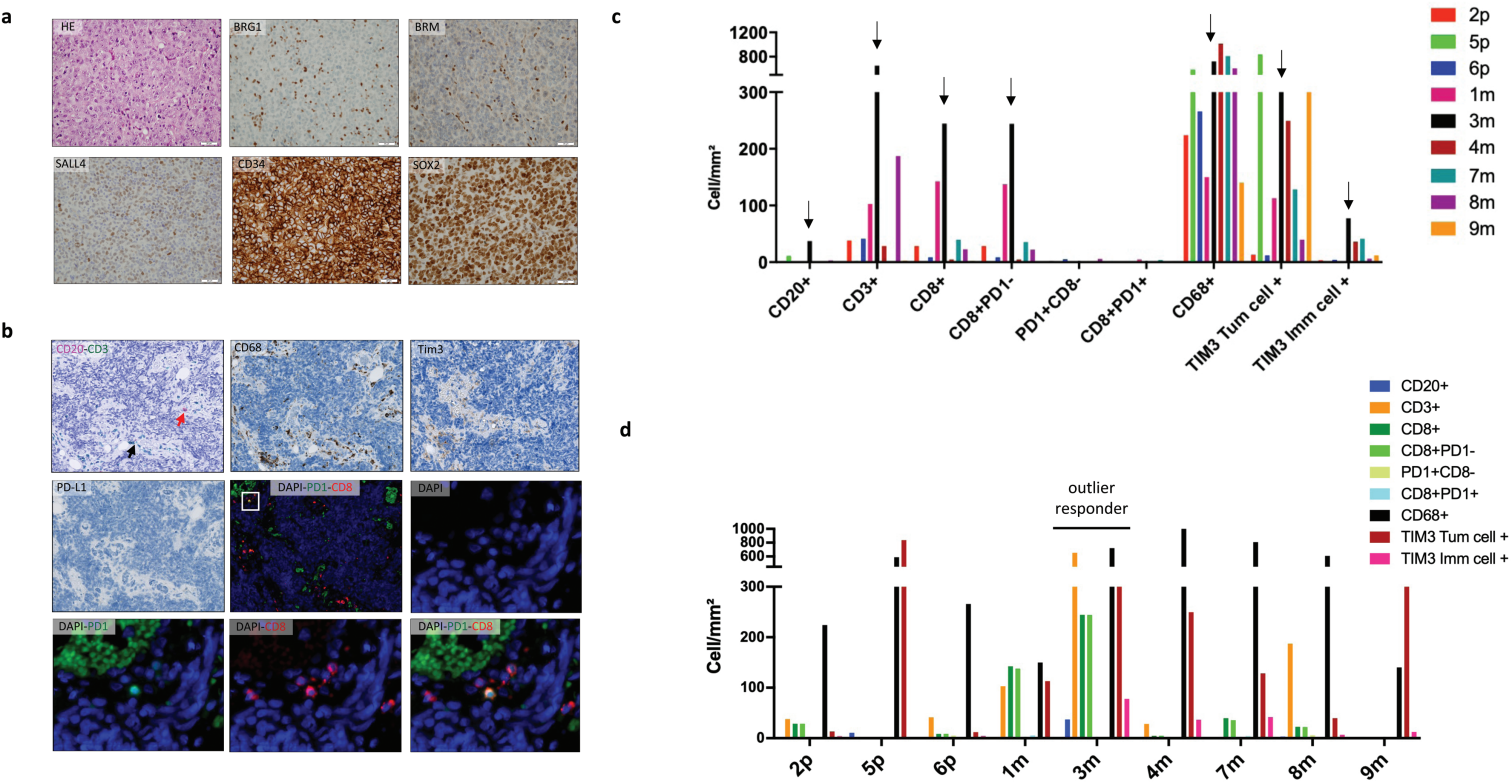
Pathologic features and immune infiltrate of SMARCA4-UT. (**a**) Hematoxylin and eosin staining showing tumor cells with epithelioid/rhabdoid features (×40). Immunohistochemical profile (×40): BRG1 and BRM, both diffusely lost in tumor cells with internal control in inflammatory cells; SALL4, focally expressed in tumor cells; CD34 and SOX2, diffusely expressed in tumor cells. (**b**) Low immune infiltrate on immunohistochemical profile: very few CD3 and CD8-positive T cells, whereas the CD68-positive macrophage cells are the most represented cell type in the TME with some TIM3-positive but PD-L1-negative tumor cells. Immunofluorescent assays: global visualization for CD8 and PD1 staining with autofluorescence from red blood cells in green, with a zoom in the very few double-positive cells in the white square. (**c**) Histogram representing immune cells densities (cells/mm^2^) shows low densities of immune infiltrate on primary tumor (p) or metastasis (m) by immune cells type (arrow indicates individual cell density for sample no. 3m from patient with the outlier response to ICI). (**d**) Histogram representing immune cells densities (cells/mm^2^) in tumor cores by specimen: primary tumors on the left and metastasis on the right. Abbreviations: SMARCA4-UT, SMARCA4-deficient thoracic sarcoma; TME, tumor microenvironment.

Pathological features were faithful to the known morphological description and “immunohistochemical signature” (Fig. 2a).^8^ Analyzed samples to assess the tumor immune infiltrate by immunostainings were those available at the diagnosis, prioritizing the primary tumor sample when possible (Fig. 2b). Eight over the 9 patients had an immune-low profile tumor with low CD3-positive T-cells density [median at 33.3 per mm^2^; range (1.1-187.3)], low CD8-positive T-cells density [median at 15.6 per mm^2^; range (0.6-142.7)], and low CD20-positive B-cells density [median at 0.3 per mm^2^; range (0-10.9)]. In these 8 tumors, CD68-positive macrophage-cells were the most represented cell type [median at 427.4 per mm^2^; range (140.4-1014.3)] and 5 out of them displayed TIM3 positivity in tumor cells (>100 cells per mm^2^). These 8 tumors were also lowly infiltrated by efficient T cells, defined by CD8-positive PD1-negative cells [median at 15.3 per mm^2^; range (0.1-137.7) and none contained any TLS; only one tumor had a weak PD-L1 expression on tumor cells (no. 7) (Fig. 2c, d). Conversely, one tumor (no. 3) was characterized by high immune cell densities (CD3+ at 654.2 per mm^2^; CD8+ at 244.4 per mm^2^, almost all PD1 negative; CD20+ at 37.3 per mm^2^), presence of TLS aggregate, and weak PD-L1 expression on both tumor cells and macrophages.

### Analysis of the Immune Infiltrate in an Independent Cohort

**Figure 3.**
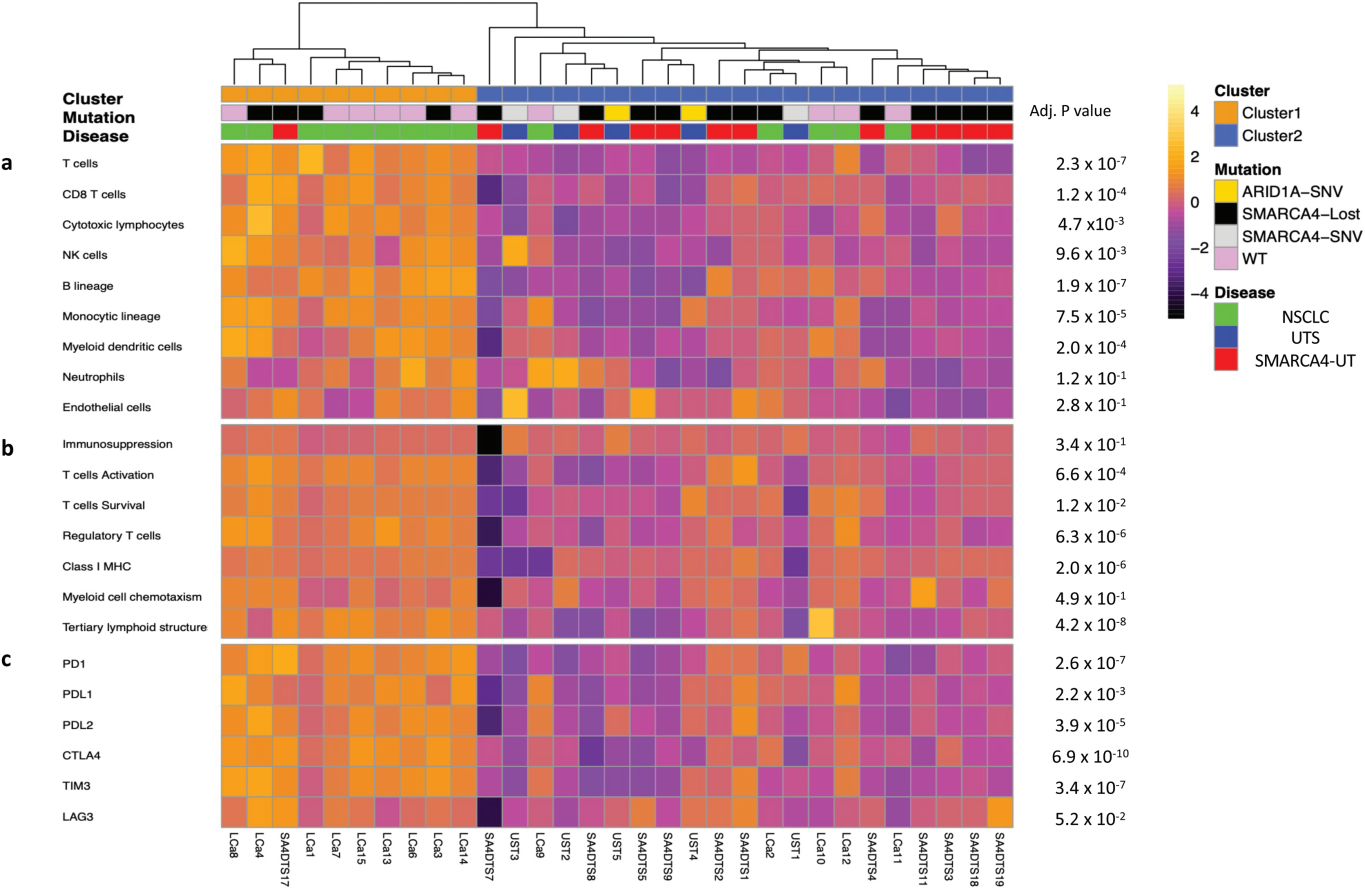
SMARCA4-UT exhibits strongly different TMEs than NOS NSCLC regardless of *SMARCA4* mutation status. This figure refers to the Le Loarer et al cohort (*n* = 31). (**a**) Unsupervised clustering of samples was performed based on the metagene Z-score for the included populations of MCP-counter. (**b**) Expression of gene signatures related to the functional orientation of the immune TME. (**c**) Expression of genes related to immune checkpoints. Abbreviations: MCP, microenvironment cell population; NK cells, natural killer cells; NOS NSCLC, not other specified non–small cell lung cancer; SMARCA4-UT, SMARCA4-deficient thoracic sarcoma; TME, tumor microenvironment; UTS: undifferentiated thoracic sarcoma.

To validate our observation, we analyzed an independent transcriptome dataset.^1^ Unsupervised hierarchical clustering using immune scores for different immune cell types identified 2 clusters namely C1 and C2; while C1 was characterized by immune rich TME, C2 was immune desert (Fig. 3a). Notably, C1 was enriched for NSCLC independently from *SMARCA4* status (*n* = 9/10; 90%) (*P* = .001), while C2 was enriched for SMARCA4-UT (*n* = 11/12; 91.7%) (*P* = .005) and UTS (*n* = 5/5; 100%) (*P* = .0005). Similarly, gene signatures associated with immunosuppression, T-cell activation and survival, regulatory T cells, major histocompatibility complex class I, and myeloid cell chemotaxis were downregulated in SMARCA4-UT and UTS relative to NSCLC (Fig. 3b). Similarly, we observed lower lymphoid-structures-associated B-cell-specific chemokine *CXCL13* in C2-enriched sarcomas cluster. Finally, we observed lower expression of immune checkpoints genes in the majority of C2-enriched sarcomas cluster relative to C1-NSCLC enriched cluster (Fig. 3c).

### Patient’s Response to Immune Checkpoint Inhibitors and Immune Infiltration Correlate

**Figure 4.**
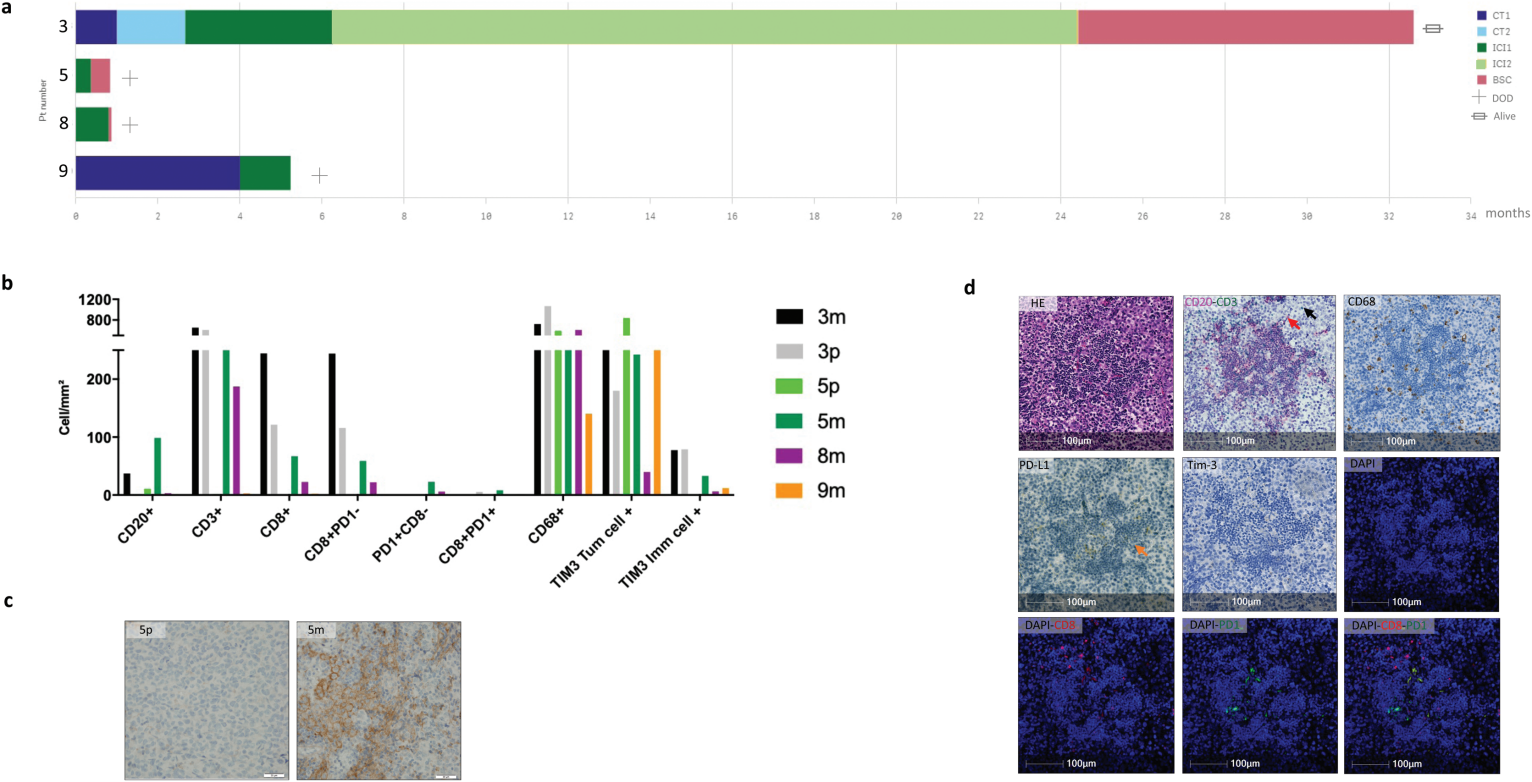
Sub-population treated by immune checkpoint inhibitor (*n* = 4). (**a**) Swimming plot of the received treatment: patient no. 3: L1: doxorubicin/ifosfamide; L2: actinomycin D/dacarbazine/cisplatin; L3: ipilimumab/nivolumab; L4 nivolumab; patient no. 5: L1: ipilimumab/nivolumab; patient no. 8: L1: nivolumab; patient no. 9: L1: carboplatin/paclitaxel; L2 nivolumab. (**b**) Immune cells densities (cells/mm^2^) in tumor cores by specimen. (**c**) PD-L1 staining on primary tumor (p) and metastasis (m) of patient no. 5. (**d**) Representation of mature TLS in the lymph node metastasis of patient no. 5 with: hematoxylin and eosin stain; IHC CD20 pink stain for B cells (red arrow) and CD3 green for T cells (black arrow); IHC CD68-positive cells for macrophages; very few PD-L1-positive cells (orange arrow); absence of TIM3-positive cells; immunofluorescent with CD8 (red) and PD1 (green) stain. Abbreviations: BSC, best supportive care; CT, chemotherapy; DOD: dead of the disease; ICI, immune checkpoint inhibitor; IHC: immunohistochemical.

Among our patients, 4 received ICI as one of their anticancer treatment. Their characteristics and outcomes are detailed in Fig. 4. Two of them (no. 5 and no. 8) died quickly within 10 weeks after the diagnosis and had ICI as their only treatment (Fig. 4a). The third one (no. 9) died within 6 months and received chemotherapy as first line and ICI as second line. Those 3 patients had immune-low tumor profile (Fig. 4b). For the patient no. 5 matched distant metastasis was also available and, in contrast with the primary tumor, weak PD-L1 expression (Fig. 4c) and mature TLS were found in the lymph node metastasis (Fig. 4d).

**Figure 5.**
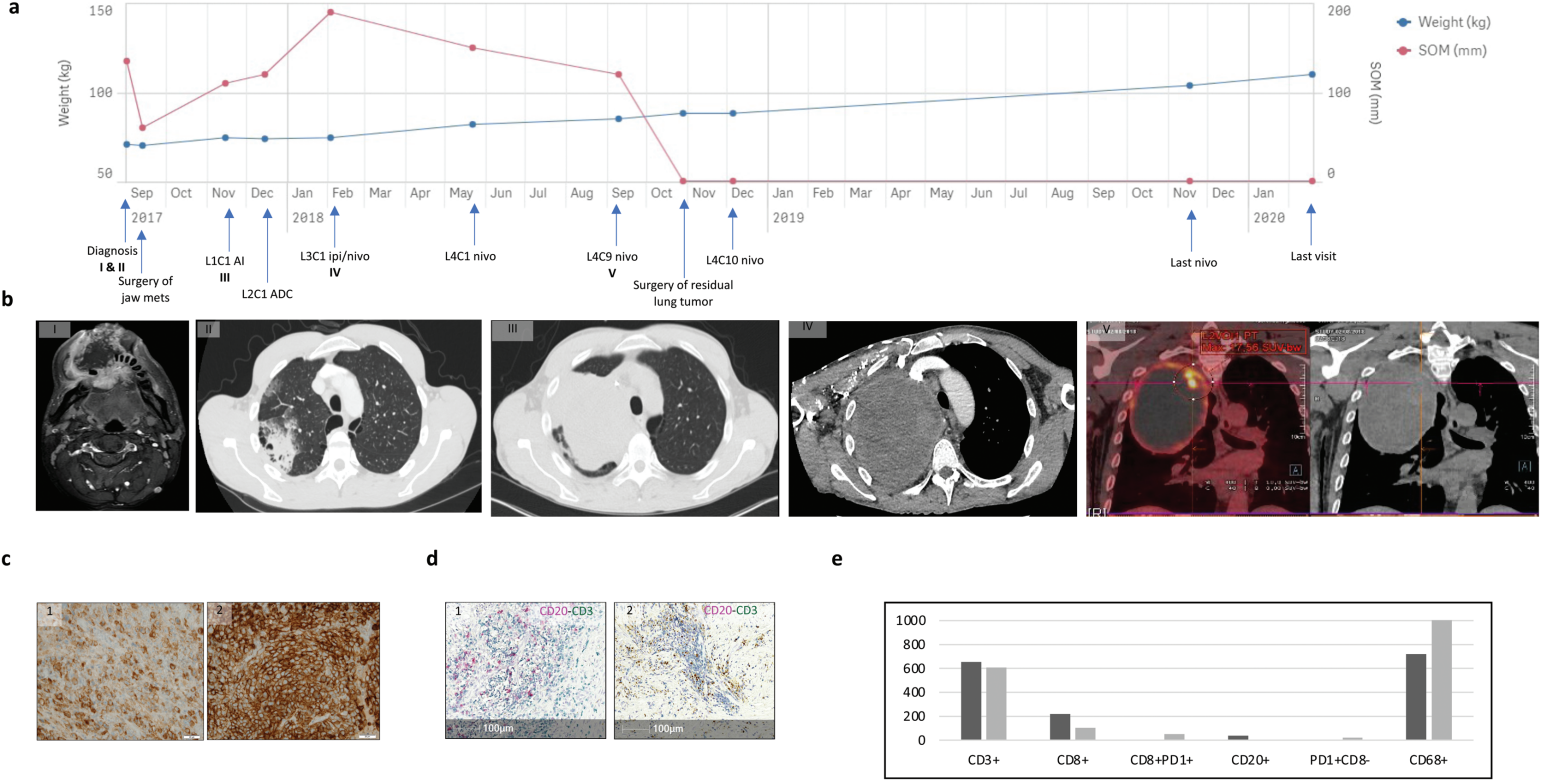
Focus on patient no. 3 with the outlier response to ICI. (**a**) Timeline of his medical history reporting the evolution of his weight and the SOM on computed tomography (CT) scans. (**b**) Images features of the evolution of the main target lesions: I: Cervical MRI-enhanced gadolinium T1-weighted axial sections of the bulky jaw metastasis; II: Thoracic CT-scan axial section showing the initial lesion in the right upper pulmonary lobe in September 2017; III: massive progression of the lesion at the beginning of the first line of chemotherapy in November 2017; IV: progressive lesion at the beginning of the ICI combination with a superior vena cava syndrome in February 2018; V: positron emission tomography-scan frontal sections showing partial response of the lesion after 5 cycles of combination of ipilimumab with nivolumab and 10 cycles of maintenance with nivolumab in September 2018, with a hypermetabolism at the periphery of the right apical solid-cystic pulmonary mass predominantly apical (SUV max 17.6). (**c**) PD-L1 IHC (×40) and (**d**) TLS presence before (1) and after ICI (2). Before ICI, one aggregate CD20 (IHC pink stain)-CD3 (IHC green stain)-positive, no longer seen after ICI (in yellow-brown, hemosiderin deposit). (**e**) Comparison of cells densities (cells/mm^2^) between tissues before (dark gray) and after (light gray) ICI. Abbreviations: ADC, actinomycin D/dacarbazine/cisplatin; AI, doxorubicin/ifosfamide; C, cycle; CT, computed tomography; IHC: immunohistochemical; ICI, immune checkpoint inhibitor; L, line; MRI, magnetic resonance imaging; SOM, sum of diameters of target lesions.

The only alive patient of the cohort was patient no. 3, a 40-year-old man who was referred for the onset of a rapidly growing mass of the lower jawbone in the setting of weight loss (Fig. 5a, b). After initial surgery of the jaw metastasis and the failure of 2 lines of chemotherapy, the patient showed rapid and partial long-term response following combination of ipilimumab and nivolumab, followed by nivolumab maintenance and surgical resection of lung residual tumor. Resection was complete leading to a complete remission status, and nivolumab was kept as maintenance treatment for almost 2 years after the initial diagnosis. Nowadays, while being on follow-up, patient is fully active and able to carry on all pre-disease performance without restriction. Regarding his tumor’s immune profile, the first tumor specimen presented a TLS aggregate even although it was not a mature one, whereas no TLS nor aggregate were observed in the second tumor specimen after several months on ICI treatment (Fig. 5c-e). Whereas CD3-positive cells density was similar in both specimens, CD20-positive cells density was higher in the specimen collected before any systemic anticancer treatment than in the residual one collected after the different treatments. We thus investigated double-positive (CD8+PD1+) cells, which showed low density in the jaw specimen, but had a tenfold increase in the residual lung tumor. In addition, PD1-positive but CD8-negative cells density was also significantly higher in the residual lung tumor compared with the jaw lesion. Puzzlingly, PD-L1 intensity staining was higher in the residual tumor. Overall, the immune infiltrate in the sample collected after ICI showed clear signs of exhaustion.

As this case was an outlier, we performed a comprehensive genomic profiling on the 2 samples collected before and after ICI. On the first sample, *SMARCA4* Q183∗ mutation was found along with *HRAS* (G13V), *TP53* (G245V), and *BLM* (D107fs∗3) mutations. Tumor was not considered as microsatellite instable and TMB profile was considered as high (29 mutations per megabase). On the second sample, no additional somatic mutation was observed. However, we observed *JAK1* and *JUN* amplification, suggesting a role of the copy number variation in genes related to IFNG pathway in resistance and immune exhaustion.

### In Vitro Analyses of knockdown of SMARCA4 in Cell Lines

**Figure 6.**
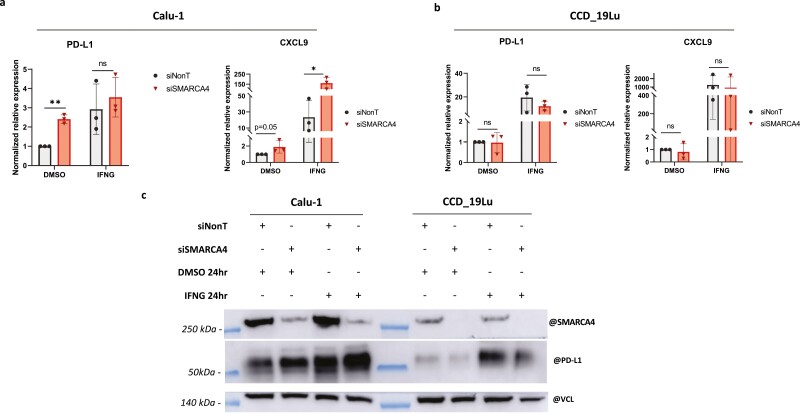
In vitro analyses on the impact of SMARCA4 loss on the response to interferon gamma treatment in 2 different cell lines (Calu-1: human lung carcinoma; CCD_19Lu: human lung fibroblasts). PD-L1 and CXCL9 chemokine relative expression in Calu-1 cell line (**a**) and in CCD_19Lu cell line (**b**). (**c**) PD-L1 protein expression according to *SMARCA4* deficiency status and interferon treatment in the 2 cell lines. Abbreviations: DMSO, dimethylsulfoxid; IFNG, interferon gamma; ns, no significate; VCL, vinculin; ∗*P* < .05,∗∗*P* < .001.

To investigate whether TME changes observed are linked to cell ontogeny, we checked in vitro if SMARCA4 loss of function could induce gene expression changes of CXCL9 chemokine and PD-L1 in NSCLC and thoracic fibroblast cell lines, treated with/without IFNG. Strikingly, we observed a significant increase of CXCL9 expression levels in NSCLC cell line following SMARCA4 knockdown with (*P* < .05) and without (*P* = .05) IFNG stimulations, while no effect was observed in the lung fibroblast cell line (Fig. 6a, b). In addition, SMARCA4 knockdown induced an upregulation of PD-L1 expression in NSCLC cell line, but only with a significant increase in the cells without IFNG stimulation (*P* < .001), with no effect in the lung fibroblast cell line (Fig. 6c).

## Discussion

To our knowledge, our study is the first one to report a comprehensive analysis of the immune infiltrate in SMARCA4-UT, by analyzing the immune cell infiltrates, the presence of TLS and PD-L1 expression in tumor and macrophage cells. Our study showed that SMARCA4-UT are mainly immune-desert tumors, differing from NSCLC but similar to most STS subtypes. This might explain limited efficacy of ICI in this setting, although we did observe one responder in our cohort.

Regardless of the tumor types, immune cell infiltrates, TLS, PD-L1 expression in tumor cells or immune infiltrating cells, TMB, IFN signature and DNA mismatch-repair deficiency status have been shown to represent potential biomarkers of response to ICI in several cancer subtypes.^40-44^ In our cohort, SMARCA4-UT without TLS-positive tumor did not respond to ICI and had a very poor OS. Among them, one patient died of local progression of his thoracic TLS-negative tumor in less than 2 months despite mature TLS in his lymph node metastasis. The outcome of this patient raises questions about the heterogeneity of the TLS status between the different tumor sites and the relevance of the evaluation of predictive factors of response to treatment at a metastatic site, while the local progression of the primary tumor is the main prognosis factor. The only surviving patient is the one who responded dramatically to ICI and had a TLS aggregate in his primary tumor. Sample from the surgery of the residual tumor after ICI showed the loss of TLS, reinforcing the correlation between TLS and response to ICI. This loss was never previously reported as a secondary resistance mechanism but might explain the clone resistant’s immune infiltrate. With this loss, immune cells could not be educated any more to recognize new tumor antigens which could thereby lead to their exhaustion. Our observation is consistent with the previous report that STS showing a class E immune-high subgroup characterized by the presence of TLS have a high response rate to ICI regardless of their histologic subtypes.^16^ The results of PEMBROSARC, the first clinical trial investigating the efficacy of ICI in TLS-positive STS, were recently presented.^45^ Briefly, TLS-positive STS were found in 48 of the 240 patients screened and among them 35 were included and 30 were eligible for efficacy. While no patient had a complete response in the TLS-positive STS, 8 patients had a partial response and 5 a stable disease. With a 6-month non-progression rate of 40% in TLS-positive STS while it was only of 4.2% in all comers, this study corroborated that TLS status is an efficient approach to tailor ICI in STS patients.

In our cohort, in addition to having TLS, the only high immune infiltrate tumor also had a high TMB which might be another explanation to the outlier response to ICI. Similarly to the change in the TLS status between samples, a *JAK1* amplification appeared on the lung residual tumor. Intriguingly, *JAK1* loss-of-function mutations or deletions have been previously described as a mechanism of ICI resistance. *JAK1/2* loss of function leads to loss of IFNG signaling, preventing PD-L1 expression and thus making ICI futile.^46^ In our case, we could hypothesize that the *JAK1* amplification might be responsible for the increased PD-L1 expression and contributed to immune cells exhaustion.

Interestingly, despite previous promising results in different cancer cohorts suggesting that BAF-deficient tumors might be more sensitive to ICI with improved clinical outcomes,^26-29,47-49^ our conflicting results among others^22-24,30^ enforced the hypothesis that wider genomic contexts than just BAF deficiency and cell’s ontogeny account for the efficacy of ICI. Indeed, in our cohort, efficacy of ICI was limited and restricted to a tumor with specific immune TME, in contrast to previous published data.^31,32,50^ The immune infiltrate does not seem to be dependent on SMARCA4-deficiency itself. Indeed, in vitro analyses reinforced the hypothesis than cell ontogeny may play a central role on the effect of *SMARCA4* loss of function. Calu-1 cell line was chosen as a NSCLC cell line, whereas the human thoracic fibroblasts cell line CCD_19Lu had to replace the non-existing SMARCA4-UT cell line. These experiments tend to show that SMARCA4 loss upregulated PD-L1 expression in the NSCLC cell line, whereas there was no difference of its expression in thoracic fibroblasts. Even after a strong induction of the IFN pathway through IFNG treatment, PD-L1 expression tends to only be slightly downregulated by SMARCA4 loss in fibroblasts. CXCL9 expression was also assessed since CXLC9 has been described as one of the chemokine reflecting the most the IFN pathway activation and has also been suggested as a predictor of ICI response.^51^ Similarly to PD-L1 expression, we did find an upregulated CXCL9 expression in the NSCLC cell line with SMARCA4 knockdown with/without IFNG stimulation, whereas there was no difference seen in the thoracic fibroblast cell line. Though the underlying mechanisms are not yet understood, SMARCA4-dependent activation of IFN signaling did differ from one cell line to the other. Moreover, in our work, immune deconvolution using an independent transcriptome dataset demonstrated a distinct immune profile between NOS NSCLC and sarcomas regardless of the *SMARCA4* mutational status. Another example is SCCOHT, previously described as immune-high tumors as opposed to SMARCA4-UT.^26,27^ Like other mutations or deficiencies, tumor cell of origin may change the driver mutation pathogenesis and clinical outcomes.

As already mentioned, in the latest edition of WHO classification of thoracic tumors, the pathology community chose to not use anymore the term “sarcoma” to described this entity, instead the term SMARCA4-UT was preferred to better reflect their possible epithelial histogenesis.^52^ In a useful way our study highlighted SMARCA4-UT similarities with other subtypes of thoracic sarcomas, but also their distinct immune profile from SMARCA4-deficient NSCLC. Therefore, these 2 tumor subtypes appear to have different characteristics, that should encourage specific research into the clinical implications of this distinction.

It is important to mention herein that our study has several limitations including the low number of cases explored, mainly due to the extreme rarity of the disease. Among the potential biomarkers of response to ICI, immune infiltrates, TLS, and PD-L1 expression were studied for all samples. However, the genomic comprehensive analysis, allowing to assess TMB and DNA mismatch-repair deficiency status, was only performed on the samples of the responder.

## Conclusion

Overall, our data demonstrate that SMARCA4-UT are mainly immune desert tumors similarly to most STS subtypes with limited efficacy to ICI. The detailed analysis of the tumor landscape of the long-term responder highlights the relevance of TLS as predictive markers to ICI efficacy in SMARCA4-UT as in STS. Finally, our data suggest that TME of SMARCA4-driven tumors varies according to the cell of origin. Further studies are needed to understand the interplay between alterations of BAF complexes, cell ontogeny and immunity.

## Supplementary Material

oyac040_suppl_Supplementary_Tables

oyac040_suppl_Supplementary_Appendix

## Data Availability

The data underlying this article will be shared on reasonable request to the corresponding author.

## References

[1] Le Loarer F , Watson S, Pierron G et al SMARCA4 inactivation defines a group of undifferentiated thoracic malignancies transcriptionally related to BAF-deficient sarcomas. Nat Genet 2015;47:1200-1 205. 26343384 10.1038/ng.3399

[2] Mashtalir N , D’Avino AR, Michel BC et al Modular organization and assembly of SWI/SNF family chromatin remodeling complexes. Cell 2018;175:1272-1288.e20. 30343899 10.1016/j.cell.2018.09.032 PMC6791824

[3] Kadoch C , Hargreaves DC, Hodges C et al Proteomic and bioinformatic analysis of mammalian SWI/SNF complexes identifies extensive roles in human malignancy. Nat Genet 2013;45:592-601. 23644491 10.1038/ng.2628 PMC3667980

[4] Shain AH , Pollack JR. The spectrum of SWI/SNF mutations, ubiquitous in human cancers. PLoS One 2013;8:e55119. 23355908 10.1371/journal.pone.0055119 PMC3552954

[5] Mittal P , Roberts CWM. The SWI/SNF complex in cancer - biology, biomarkers and therapy. Nat Rev Clin Oncol 2020;17:435-448. 32303701 10.1038/s41571-020-0357-3 PMC8723792

[6] Tsao M. PL01.05 The new WHO classification of lung tumors. J Thorac Oncol 2021;16:S63.

[7] Crombé A , Alberti N, Villard N et al Imaging features of SMARCA4-deficient thoracic sarcomas: a multi-centric study of 21 patients. Eur Radiol 2019. https://doi.org/10.1007/s00330-019-06017-x. 10.1007/s00330-019-06017-x 30762113

[8] Perret R , Chalabreysse L, Watson S et al SMARCA4-deficient thoracic sarcomas: clinicopathologic study of 30 cases with an emphasis on their nosology and differential diagnoses. Am J Surg Pathol 2019;43:455-465. 30451731 10.1097/PAS.0000000000001188

[9] Sauter JL , Graham RP, Larsen BT et al SMARCA4-deficient thoracic sarcoma: a distinctive clinicopathological entity with undifferentiated rhabdoid morphology and aggressive behavior. Mod Pathol Off J U S Can Acad Pathol Inc 2017;30:1422-1432. 10.1038/modpathol.2017.61 28643792

[10] Yoshida A , Kobayashi E, Kubo T et al Clinicopathological and molecular characterization of SMARCA4-deficient thoracic sarcomas with comparison to potentially related entities. Mod Pathol Off J U S Can Acad Pathol Inc 2017;30:797-809. 10.1038/modpathol.2017.11 28256572

[11] Takeda M , Tani Y, Saijo N et al Cytopathological features of SMARCA4-deficient thoracic sarcoma: report of 2 cases and review of the literature. Int J Surg Pathol 2020;28:109-114. 31448657 10.1177/1066896919870866

[12] Groisberg R , Hong DS, Behrang A et al Characteristics and outcomes of patients with advanced sarcoma enrolled in early phase immunotherapy trials. J Immunother Cancer 2017;5:100. 29254498 10.1186/s40425-017-0301-y PMC5735899

[13] Tawbi HA , Burgess M, Bolejack V et al Pembrolizumab in advanced soft-tissue sarcoma and bone sarcoma (SARC028): a multicentre, two-cohort, single-arm, open-label, phase 2 trial. Lancet Oncol 2017. https://doi.org/10.1016/S1470-2045(17)30624-1. 10.1016/S1470-2045(17)30624-1 PMC7939029 28988646

[14] Toulmonde M , Penel N, Adam J et al Use of PD-1 Targeting, macrophage infiltration, and ido pathway activation in sarcomas: A phase 2 clinical trial. JAMA Oncol 2017. https://doi.org/10.1001/jamaoncol.2017.1617. 10.1001/jamaoncol.2017.1617 PMC5833654 28662235

[15] Blay J-Y , Penel N, Ray-Coquard IL et al High clinical activity of pembrolizumab in chordoma, alveolar soft part sarcoma (ASPS) and other rare sarcoma histotypes: The French AcSé pembrolizumab study from Unicancer. J Clin Oncol 2021;39:11520-11520.

[16] Petitprez F , de Reyniès A, Keung EZ et al B cells are associated with survival and immunotherapy response in sarcoma. Nature 2020. https://doi.org/10.1038/s41586-019-1906-8. 10.1038/s41586-019-1906-8 31942077

[17] Dieu-Nosjean M-C , Goc J, Giraldo NA et al Tertiary lymphoid structures in cancer and beyond. Trends Immunol 2014;35:571-580. 25443495 10.1016/j.it.2014.09.006

[18] Engelhard VH , Rodriguez AB, Mauldin IS et al Immune cell infiltration and tertiary lymphoid structures as determinants of antitumor immunity. J Immunol Baltim MD 1950. 2018;200:432-442. 10.4049/jimmunol.1701269 PMC5777336 29311385

[19] Sautès-Fridman C , Petitprez F, Calderaro J et al Tertiary lymphoid structures in the era of cancer immunotherapy. Nat Rev Cancer 2019;19:307-325. 31092904 10.1038/s41568-019-0144-6

[20] Keung EZ , Burgess M, Salazar R et al Correlative analyses of the SARC028 trial reveal an association between sarcoma-associated immune infiltrate and response to pembrolizumab. Clin Cancer Res Off J Am Assoc Cancer Res 2020. https://doi.org/10.1158/1078-0432.CCR-19-1824. 10.1158/1078-0432.CCR-19-1824 PMC7731262 31900276

[21] Zhou M , Yuan J, Deng Y et al Emerging role of SWI/SNF complex deficiency as a target of immune checkpoint blockade in human cancers. Oncogenesis 2021;10:3. 33419967 10.1038/s41389-020-00296-6 PMC7794300

[22] Abou Alaiwi S , Nassar AH, Xie W et al Mammalian SWI/SNF complex genomic alterations and immune checkpoint blockade in solid tumors. Cancer Immunol Res 2020. https://doi.org/10.1158/2326-6066.CIR-19-0866. 10.1158/2326-6066.CIR-19-0866 PMC7415546 32321774

[23] Li J , Wang W, Zhang Y et al Epigenetic driver mutations in ARID1A shape cancer immune phenotype and immunotherapy. J Clin Invest 2020;130:2712-2726. 32027624 10.1172/JCI134402 PMC7190935

[24] Liu X-D , Kong W, Peterson CB et al PBRM1 loss defines a nonimmunogenic tumor phenotype associated with checkpoint inhibitor resistance in renal carcinoma. Nat Commun 2020;11:2135. 32358509 10.1038/s41467-020-15959-6 PMC7195420

[25] Leruste A , Chauvin C, Pouponnot C et al Immune responses in genomically simple SWI/SNF-deficient cancers. Cancer 2021;127:172-180. 33079397 10.1002/cncr.33172

[26] Lu B , Shi H. An in-depth look at small cell carcinoma of the ovary, hypercalcemic type (SCCOHT): clinical implications from recent molecular findings. J Cancer 2019;10:223-237. 30662543 10.7150/jca.26978 PMC6329856

[27] Jelinic P , Ricca J, Van Oudenhove E et al Immune-active microenvironment in small cell carcinoma of the ovary, hypercalcemic type: rationale for immune checkpoint blockade. J Natl Cancer Inst 2018;110:787-790. 29365144 10.1093/jnci/djx277 PMC6037122

[28] Hanna GJ , Lizotte P, Cavanaugh M et al Frameshift events predict anti-PD-1/L1 response in head and neck cancer. JCI Insight 2018;3. https://doi.org/10.1172/jci.insight.98811. 10.1172/jci.insight.98811 PMC5916245 29467336

[29] Schoenfeld AJ , Bandlamudi C, Lavery JA et al the genomic landscape of SMARCA4 alterations and associations with outcomes in patients with lung cancer. Clin Cancer Res Off J Am Assoc Cancer Res 2020;26:5701-5708. 10.1158/1078-0432.CCR-20-1825 PMC7641983 32709715

[30] Marinelli D , Mazzotta M, Scalera S et al KEAP1-driven co-mutations in lung adenocarcinoma unresponsive to immunotherapy despite high tumor mutational burden. Ann Oncol Off J Eur Soc Med Oncol 2020;31:1746-1754. 10.1016/j.annonc.2020.08.2105 32866624

[31] Henon C , Blay J-Y, Massard C et al Long lasting major response to pembrolizumab in a thoracic malignant rhabdoid-like SMARCA4-deficient tumor. Ann Oncol Off J Eur Soc Med Oncol 2019;30:1401-1403. 10.1093/annonc/mdz160 31114851

[32] Takada K , Sugita S, Murase K et al Exceptionally rapid response to pembrolizumab in a SMARCA4-deficient thoracic sarcoma overexpressing PD-L1: A case report. Thorac Cancer 2019;10:2312-2315. 31617320 10.1111/1759-7714.13215 PMC6885443

[33] Anžič N , Krasniqi F, Eberhardt A-L et al Ipilimumab and pembrolizumab mixed response in a 41-year-old patient with SMARCA4-deficient thoracic sarcoma: an interdisciplinary case study. Case Rep Oncol 2021;14:706-715. 34177520 10.1159/000515416 PMC8215992

[34] Kawachi H , Kunimasa K, Kukita Y et al Atezolizumab with bevacizumab, paclitaxel and carboplatin was effective for patients with SMARCA4-deficient thoracic sarcoma. Immunotherapy 2021;13:799-806. 34030451 10.2217/imt-2020-0311

[35] Calderaro J , Petitprez F, Becht E et al Intra-tumoral tertiary lymphoid structures are associated with a low risk of early recurrence of hepatocellular carcinoma. J Hepatol. 2019;70:58-65. 30213589 10.1016/j.jhep.2018.09.003

[36] Vanhersecke L , Brunet M, Guégan J-P et al Mature tertiary lymphoid structures predict immune checkpoint inhibitor efficacy in solid tumors independently of PD-L1 expression. Nat Cancer 2021;2:794-802. 35118423 10.1038/s43018-021-00232-6 PMC8809887

[37] Dobin A , Davis CA, Schlesinger F et al STAR: ultrafast universal RNA-seq aligner. Bioinforma Oxf Engl 2013;29:15-21. 10.1093/bioinformatics/bts635 PMC3530905 23104886

[38] Li B , Dewey CN. RSEM: accurate transcript quantification from RNA-Seq data with or without a reference genome. BMC Bioinformatics 2011;12:323. 21816040 10.1186/1471-2105-12-323 PMC3163565

[39] Becht E , Giraldo NA, Lacroix L et al Estimating the population abundance of tissue-infiltrating immune and stromal cell populations using gene expression. Genome Biol 2016;17:218. 27765066 10.1186/s13059-016-1070-5 PMC5073889

[40] Duffy MJ , Crown J. Biomarkers for predicting response to immunotherapy with immune checkpoint inhibitors in cancer patients. Clin Chem 2019;65:1228-1238. 31315901 10.1373/clinchem.2019.303644

[41] Lapuente-Santana Ó , Eduati F. Toward systems biomarkers of response to immune checkpoint blockers. Front Oncol 2020;10:1027. 32670886 10.3389/fonc.2020.01027 PMC7326813

[42] Walk EE , Yohe SL, Beckman A et al The cancer immunotherapy biomarker testing landscape. Arch Pathol Lab Med 2020;144:706-724. 31714809 10.5858/arpa.2018-0584-CP

[43] Jardim DL , Goodman A, de Melo Gagliato D et al The challenges of tumor mutational burden as an immunotherapy biomarker. Cancer Cell 2021;39:154-173. 33125859 10.1016/j.ccell.2020.10.001 PMC7878292

[44] Paijens ST , Vledder A, de Bruyn M et al Tumor-infiltrating lymphocytes in the immunotherapy era. Cell Mol Immunol 2021;18:842-859. 33139907 10.1038/s41423-020-00565-9 PMC8115290

[45] Italiano A , Bessede A, Bompas E et al PD1 inhibition in soft-tissue sarcomas with tertiary lymphoid structures: a multicenter phase II trial. J Clin Oncol 2021;39:11507-11507.

[46] Shin DS , Zaretsky JM, Escuin-Ordinas H et al Primary resistance to PD-1 blockade mediated by JAK1/2 mutations. Cancer Discov 2017;7:188-201. 27903500 10.1158/2159-8290.CD-16-1223 PMC5296316

[47] Miao D , Margolis CA, Gao W et al Genomic correlates of response to immune checkpoint therapies in clear cell renal cell carcinoma. Science 2018;359:801-806. 29301960 10.1126/science.aan5951 PMC6035749

[48] Braun DA , Ishii Y, Walsh AM et al Clinical validation of PBRM1 alterations as a marker of immune checkpoint inhibitor response in renal cell carcinoma. JAMA Oncol 2019. https://doi.org/10.1001/jamaoncol.2019.3158. 10.1001/jamaoncol.2019.3158 PMC6735411 31486842

[49] Li L , Li M, Jiang Z et al ARID1A mutations are associated with increased immune activity in gastrointestinal cancer. Cells 2019;8. https://doi.org/10.3390/cells8070678. 10.3390/cells8070678 PMC6678467 31277418

[50] ESMO. Pembrolizumab Delivers Clinical Benefit in Selected Histotypes of Rare Sarcoma. Available at https://www.esmo.org/oncology-news/pembrolizumab-delivers-clinical-benefit-in-selected-histotypes-of-rare-sarcoma Accessed March 1, 2021.

[51] Litchfield K , Reading JL, Puttick C et al Meta-analysis of tumor- and T cell-intrinsic mechanisms of sensitization to checkpoint inhibition. Cell 2021;184:596-614.e14. 33508232 10.1016/j.cell.2021.01.002 PMC7933824

[52] Rekhtman N , Montecalvo J, Chang JC et al SMARCA4-deficient thoracic sarcomatoid tumors represent primarily smoking-related undifferentiated carcinomas rather than primary thoracic sarcomas. J Thorac Oncol Off Publ Int Assoc Study Lung Cancer 2020;15:231-247. 10.1016/j.jtho.2019.10.023 PMC7556987 31751681

