# Supplementary material for: Immune-Desert Tumor Microenvironment in Thoracic SMARCA4-Deficient Undifferentiated Tumors with Limited Efficacy of Immune Checkpoint Inhibitors
Source: Oncologist. 2022 Mar 12;27(6):501–11. doi: 10.1093/oncolo/oyac040 (PMC9177113; doi:10.1093/oncolo/oyac040)
Supplement: oyac040_suppl_Supplementary_Tables [file oyac040_suppl_supplementary_tables.docx]

**Supplementary Table 1.** Antibodies used for immunohistochemistry on diagnosis purposes.

| **Antibody** | **References** | **Species** | **Clone** | **Concentration (µg/mL)** | **Antigen Retrieval** | **Secondary Antibody** | **Tertiary Reagents** |
| --- | --- | --- | --- | --- | --- | --- | --- |
| pankeratin | MM France/Cell marque/ F313M-16 | Mouse | AE1-AE3 | - | pH 8-8.5 | Ultraview multimer Immunoglobulin  ultraview universal DAB detection kit, Ventana, Roche Tissue Diagnostics | Ultraview DAB  ultraview universal DAB detection kit, Ventana, Roche Tissue Diagnostics |
| TTF1 | Diagomic's / Zytomed /MSK004-05 | Mouse | 8G7G3/1 | - |  |  |  |
| P40 | Clinisciences/ DBS peasanton/ RP163-05 | Rabbit | Polyclonal | - |  |  |  |
| SMARCA4/BRG1 | Abcam / 110641 | Rabbit | EPNCIR111A | - |  |  |  |
| SMARCA2/BRM | Ozyme/cell signaling / 11966S | Rabbit | D9E8B | 0.67 |  |  |  |
| CD34 | Cliniscience/ emergoeurope/ Mob098 | Mouse | QBEnd10 | - |  |  |  |
| SALL4 | Roche/ cell marque/ 760-4864 | Mouse | 6E3 | 0.47 |  |  |  |
| SOX2 | Roche/cell marque/ 760-4621 | Rabbit | SP76 | 0.03 |  |  |  |
| NUT | Ozyme/ cell signaling/3625 | Rabbit | C52B1 | 2 |  |  |  |

**Supplementary Table 2.** Antibodies used for immunohistochemistry and immunofluorescence to assess the tumors immune infiltrate.

| **Antibody** | **References** | **Species** | **Clone** | **Concentration (µg/mL)** | **Antigen Retrieval** | **Secondary Antibody** | **Tertiary Reagents** |
| --- | --- | --- | --- | --- | --- | --- | --- |
| CD3 | Agilent / A0452 | Rabbit | NA | 7,5 | pH 6 | EnVision+ System-HRP, Labelled Polymer (Rabbit) | Permanent HRP Green |
| CD8 | Agilent/ M7103 | Mouse IgG1 | C8/144B | 1.57 | pH9 | EnVision+ System-HRP, Labelled Polymer (Mouse) | Alexa Fluor^TM^ 647 Tyramide Reagent |
| CD20 | Agilent/ M0755 | Mouse IgG2a | L26 | 0.6 | pH6 | Polyview Plus AP (anti-mouse) reagent | HighDef red IHC chromogen (AP) |
| CD68 | Agilent/ M0876 | Mouse IgG3 | PG-M1 | 0.3 | pH 6 | EnVision+ System-HRP, Labelled Polymer (Mouse) | DAB |
| PD-1 | C signaling/ 43248 | Mouse IgG2a | EH33 | 1.5 | pH6 | EnVision+ System-HRP, Labelled Polymer (Mouse) | Alexa Fluor^TM^ 555 Tyramide Reagent |
| PD-L1 | abcam / ab228462 | Rabbit | SP142 | 0.2 | pH9 | EnVision+ System-HRP, Labelled Polymer (Rabbit) | DAB |
| TIM-3 | R&D / MAB23652-100 | Rabbit | 2321C | 5 | Ph6 | Polyview Plus HRP (anti-rabbit) reagent | DAB |
