# Supplementary material for: Immune-Desert Tumor Microenvironment in Thoracic SMARCA4-Deficient Undifferentiated Tumors with Limited Efficacy of Immune Checkpoint Inhibitors
Source: Oncologist. 2022 Mar 12;27(6):501–11. doi: 10.1093/oncolo/oyac040 (PMC9177113; doi:10.1093/oncolo/oyac040)
Supplement: oyac040_suppl_Supplementary_Appendix [file oyac040_suppl_supplementary_appendix.docx]

**­­­Supplemental Appendix**

**Materials and Methods**

**Immunohistochemistry and immunofluorescence**

Immunostainings carried out on diagnostic purposes were performed by the local expert sarcoma pathologist, according to routine practice (Supplementary Table 1).

Immunostainings were done on 4-μm thick tumor sections from formalin-fixed paraffin-embedded blocks (Supplementary Table 2). Antigen retrieval (Agilent, Target Retrieval Solution) of deparaffinized and rehydrated sections was performed at pH6.1 (EnVision FLEX Target Retrieval Solution, Low pH 50x concentrated Citrate buffer) for CD3, CD20, CD68, PD1, and TIM3 or at pH9 (EnVision FLEX Target Retrieval Solution, High pH 50x concentrated Tris/EDTA buffer) for CD8 and PD-L1. Secondary antibodies were revelated by 3,3′-diaminobenzidine (Dako, K3468 DAB) for CD68, PD-L1, and TIM3, High-Def red IHC chromogen (AP) (Enzo, ADI-950-140-0030) for CD20 and Permanent HRP Green (Zytomed Systems, ZUC070-100) for CD3. Endogenous Peroxidase block (H2O2 3% Gifrer, 1060351) and Protein Block (Dako, X0909) reagents were used for endogenous peroxidase, alkaline phosphatase and FcR blocking. Immunohistochemistry was processed on an automated immunostainer (AutostainerPlusLink 48, Dako). The nuclei were counterstained with hematoxylin (Dako, S3301) and slides were scanned with a Nanozoomer (Hamamatsu) after mounting with Glycergel Mounting Medium (Dako, C056330-2).

Fluorescent multiplexed immunohistochemistry was performed to detect CD8 and PD-1, using Tyramide system amplification (TSA) (TSA AF555 for PD-1 and TSA AF647 for CD8 at 1:200 dilution) after incubation with horseradish peroxidase (HRP)-conjugated polymer. Antibody stripping was performed for 97°C for 10 min. The nuclei were stained with DAPI Solution (Thermo Fisher, 62248) at 2 μg.ml^−1^ for 10 min. After mounting with ProLongTM Gold Antifade Mountant (Thermofisher, P36934), the slides were scanned at 40X magnification using a Zeiss Axio scan.Z1 device. Immune cells density (cells number/mm^2^) and TLS were quantified in the tumoral cores with Halo10 software (Indica labs).

**Gene expression analysis**

The gene expression analysis of public data was made on SMARCA4-UT (n=12), SMARCA4-deficient NSCLC (n=4), NOS NSCLC (n=10), and unclassified thoracic sarcoma (UTS) (n=5) from the Le Loarer et al. dataset ^1^. The UTS corresponded to the five cases of thoracic sarcomas that were not SMARCA4-deficient, derived from the initial cohort of unclassified sarcomas on which they worked to find new fusion transcripts or mutations. No more description was provided about these UTS in the original publication, apart from the fact that they did not cluster with SMARCA4-UT or NSCLC based on transcriptomic data, but with other unclassified sarcomas regardless of primary location.
